# Mitochondrial membrane models built from native lipid extracts: Interfacial and transport properties

**DOI:** 10.3389/fmolb.2022.910936

**Published:** 2022-09-23

**Authors:** Olivia Schiaffarino, David Valdivieso González, Inés M. García-Pérez, Daniel A. Peñalva, Víctor G. Almendro-Vedia, Paolo Natale, Iván López-Montero

**Affiliations:** ^1^ Departamento Química Física, Universidad Complutense de Madrid, Madrid, Spain; ^2^ Instituto de Investigación Biomédica Hospital Doce de Octubre (imas12), Madrid, Spain; ^3^ Instituto de Investigaciones Bioquímicas de Bahía Blanca (INIBIBB), ConsejoNacional de Investigaciones Científicas y Técnicas (CONICET), Universidad Nacional del Sur (UNS), Bahía Blanca, Argentina; ^4^ Departamento de Biología, Bioquímica y Farmacia, Universidad Nacional del Sur (UNS), Bahía Blanca, Argentina; ^5^ Departamento de Farmacia Galénica y Tecnología Alimentaria, Universidad Complutense de Madrid, Madrid, Spain; ^6^ Instituto Pluridisciplinar, Universidad Complutense de Madrid, Madrid, Spain

**Keywords:** outer mitochondrial membrane, inner mitochondrial membrane, langmuir monolayers, supported lipid bilayers, giant unilamellar vesicles (GUV), FRAP, micropipettes

## Abstract

The mitochondrion is an essential organelle enclosed by two membranes whose functionalities depend on their very specific protein and lipid compositions. Proteins from the outer mitochondrial membrane (OMM) are specialized in mitochondrial dynamics and mitophagy, whereas proteins of the inner mitochondrial membrane (IMM) have dedicated functions in cellular respiration and apoptosis. As for lipids, the OMM is enriched in glycerophosphatidyl choline but cardiolipin is exclusively found within the IMM. Though the lipid topology and distribution of the OMM and IMM are known since more than four decades, little is known about the interfacial and dynamic properties of the IMM and OMM lipid extracts. Here we build monolayers, supported bilayers and giant unilamellar vesicles (GUVs) of native OMM and IMM lipids extracts from porcine heart. Additionally, we perform a comparative analysis on the interfacial, phase immiscibility and mechanical properties of both types of extract. Our results show that IMM lipids form more expanded and softer membranes than OMM lipids, allowing a better understanding of the physicochemical and biophysical properties of mitochondrial membranes.

## Introduction

Biological membranes are key to cellular organization and compartmentalization of organelles inside the eukaryotic cell. Mitochondria are large, rod-shaped organelles central to the cellular metabolism responsible to produce cellular energy in form of adenosine triphosphate (ATP) *via* the oxidative phosphorylation (OXPHOS) or aerobic respiration through the oxidation of pyruvate to carbon dioxide and water (Sousa et al., 2018). Mitochondria occupy up to 25% of the volume of the cellular cytoplasm, where they form a highly dynamic network that continuously undergoes cycles of fusion and fission (Giacomello et al., 2020). The mitochondrial ultrastructure shows that the organelle is engulfed by two phospholipid membrane bilayers: the outer mitochondrial membrane (OMM) and the inner mitochondrial membrane (IMM) (Palade, 1953). Both membranes are separated by the mitochondrial intermembrane space (IMS) and the highly invaginated IMM encloses the mitochondrial lumen or matrix that predominantly contains proteins involved in carbon metabolism of the cell (Srere and Sumegi, 1986). The lipid composition of both membrane bilayers differs. The OMM resembles the eukaryotic plasma membrane that is mainly composed of phosphatidylcholine (PC), phosphatidylinositol (PI), sphingomyelin (SM), and cholesterol (chol) whereas the IMM shares a high similarity to the bacterial membrane that mainly contains phosphatidylethanolamine (PE), phospatidylglycerol (PG), and cardiolipin (CL) (Horvath and Daum, 2013). Within the OMM or IMM bilayers, the lipids distribution is not random and a combination of lipids with different topological and mechanical characteristics modulate the fluidity, the permeability or the mechanical strength (Dowhan, 1997). The OMM forms a smooth enriched lipid surface with very high fluidity, whereas the invaginated IMM displays a rough surface due to its elevated protein-to-lipid ratio when compared to the outer membrane (Comte et al., 1976; Horvath and Daum, 2013). The maintenance of the specific lipid bilayer composition mainly relies on the lipid metabolism including the *de novo* synthesis or the recycling form the diet. The presence of particular lipids regulates the sorting, the formation, and the function of oligomeric protein complexes (Coskun and Simons, 2011). The catalytic activities of integral or peripherally anchored proteins depend on their native structures and strongly influenced by the surrounding lipid arrangement in the lipid bilayer (Coskun and Simons, 2011). Moreover, it was shown that changes in phospholipid composition affect mitochondrial respiration, which on its turn is linked apoptosis (Crimi and Esposti, 2011), Barth syndrome (Schlame and Ren, 2009), ischemia (Brown et al., 2013), or heart failure (El-Hafidi et al., 2020).

Despite of the large amount of research on the organization dynamics of mitochondrial membranes (Yang et al., 2021), the interfacial characterization of natural OMM and IMM lipid extracts were not explored so far. The interfacial properties of lipid membranes play an important role in the stability, permeability and deformability of cells or artificial liposomal systems. Currently, most experimental setups found in the literature on the mechanical properties of the mitochondrial membranes were performed using defined membrane models with controlled formulations that mimic the IMM lipid composition with a focus on individual lipids. In particular, CL was proposed to act as a modulator of the mechanical properties of the IMM lipid bilayer (Nichols-Smith et al., 2004; Sennato et al., 2005; Domenech et al., 2006; Khalifat et al., 2008) and shown to strongly affect the thermodynamic and mechanical properties of the IMM bilayer suggesting a reduction of the energy required for *cristae* formation (Schlame and Ren, 2009). Also, coarse-grained (CG) simulations of CL-containing membranes focused on the CL reorganization properties and the lipid-protein interactions (Róg et al., 2009; Boyd et al., 2017; Boyd et al., 2018; Wilson et al., 2019; Corey et al., 2021). Lipids adopt a near cylindrical shape in mimicking mitochondrial membranes (Daum and Vance, 1997) composed of POPC, DOPE, and CL at increasing concentrations varying from 5 to 20 mol% (Wilson et al., 2019). However, local surface deviations bent into a negative curvature, also known as negative buckling that may be the underlying mechanism for the reorganization of CL into the negatively curved regions of natural membranes, a phenomenon that can experimentally be appreciated for natural CL-containing lipid membranes (Mileykovskaya and Dowhan, 2009) or nanotubes (Beltrán-Heredia et al., 2019). Though the bilayer thickness was not altered by the CL concentration, a relative decrease in lateral lipid diffusion was observed at concentrations below 10 mol% of CL that could be restored at a CL concentration of 15 mol% (Wilson et al., 2019). This non-monotonic behaviour has been reported experimentally (Unsay et al., 2013). The physiological CL concentration is then crucial for optimal membrane properties.

CL-protein interactions where shown to be mainly ruled through specific binding patches typically composed of positively charged residues (arginine and lysine) or non-polar residues (leucine, isoleucine, and valine), respectively (Planas-Iglesias et al., 2015). Similar results were obtained more recently for CL-containing cytoplasmic membranes of the Gram-negative bacteria *Escherichia coli* (Corey et al., 2021). Their results show a strong bilayer asymmetry with an enriched proportion of anionic lipid in the inner membrane leaflet of the membrane that faces the bacterial cytoplasm.

In addition, with the recent interest in mitochondrial dysfunction, there is also increasing research in the mechanical properties of OMM lipid bilayer during mitochondrial fusion and fission (Bramkamp, 2018; Daste et al., 2018). CG membrane models of the OMM in the absence of proteins have been simulated to correlate the changes of local lipid environments with conformational changes of the entire OMM (Pezeshkian et al., 2020). Analogous to the IMM, CG models of OMM or the entire mitochondrion needs to incorporate specific protein systems to probe the membrane remodelling throughout mitochondrial function and maintenance.

Here, we present a first approximation to get insights to the interfacial properties of isolated native OMM and IMM lipid extracts obtained from porcine heart mitochondria. OMM and IMM were isolated without the use of detergents through a combination of differential centrifugation and sucrose density gradients (Comte and Gautheron, 1979). The isolated lipid extracts were then tested for interfacial properties using lipid monolayers, supported bilayers (SLB), or lipid vesicles built as giant unilamellar vesicles (GUVs) in the cell-size scale. The surfactant behaviour of OMM and IMM lipids was comparatively examined through surface tension measurements at the air-water interface. Moreover, Fluorescence Recovery After Photobleaching (FRAP) allowed us to determine the lipid lateral diffusion coefficients within OMM and IMM lipid supported bilayers. We also visualized the phase separation of lipids and the presence of lipid domains in giant unilamellar vesicles of the OMM lipid extract (OMM-GUVs) through fluorescence confocal microscopy. Finally, the compression of Langmuir monolayers of the OMM and IMM lipid extracts and micropipette aspiration experiments of giant unilamellar vesicles allowed us to measure the area compressibility and the bending moduli, respectively, of both the OMM and IMM lipid extracts (Longo and Ly, 2007).

Both the mechanical and diffusion properties of lipid membranes play an important role in numerous cellular and physiological processes. The ability of biomembranes to deform is crucial for membrane fission during cell division, membrane fusion in viral infection or the flow of red blood cells in the bloodstream. Likewise, membrane fluidity modulates the activity of many important proteins in biological membranes. As the properties of a lipid bilayer are governed by lipid acyl chain length, headgroup type, and the presence of membrane proteins, a comparative analysis of lipid extracts has significant implications for membrane biology studies. Our results show a difference in the membrane stiffness and phase behaviour of OMM and IMM lipids compatible with the different lipid composition of both lipid extracts. Overall, the OMM lipid extract forms stiffer and more condensed model membranes than IMM lipids and present micron-scale phase immiscibility. OMM and IMM lipid extracts can be used in future multiple protein-lipid interaction studies where their physicochemical characterization can provide a better understanding of the biophysical properties of mitochondrial membranes.

## Materials and methods

### Chemicals

Except from the extracted mitochondrial lipids used in this work, we purchased lipids 1-palmitoyl-2-oleoyl-glycero-3-phosphocholine (PC), 1,2-dioleoyl-sn-glycero-3-phosphoethanolamine (PE), bovine brain sphingomyelin (SM) and bovine heart cardiolipin (CL), 1-palmitoyl-2-oleoyl-sn-glycero-3-phosphoinositol (PI), 1-palmitoyl-2-oleoyl-sn-glycero-3-phospho-L-serine (PS), 1,2-dioleoyl-sn-glycero-3-phosphoethanolamine-N-(lissamine rhodamine B sulfonyl) (RhPE) and cholesterol (Chol) from Avanti Polar Lipids (Alabaster, AL, United States).

### Isolation and separation of the outer and inner mitochondrial membranes

The isolation and separation of the outer (OMM) and inner mitochondrial membranes (IMMs) is based on mitochondrial rupture in the absence of detergents by swelling in the presence of inorganic phosphate, physical rupture by shear stress and the use of discontinuous sucrose gradients. First, intact mitochondria were isolated from fresh homogenized fresh pig heart (Slaughterhouse Toledo, Spain) in 0.25 M sucrose, 10 mM Tris-HCl (pH 7.4) and washed twice in the same medium before fragmentation as previously described (Smith, 1967). The washed mitochondrial pellet (20 mg protein/mL) is homogenized at 0°C–4°C in a Potter-Elvehjem homogenizer with a loose-fitting pestle in 10 mM potassium phosphate (pH 7.4) and then diluted in the same medium to a final concentration of 1 mg protein per ml and allowed to swell for 20 min. In addition, the mitochondria were broken physically by shear stress with a 1 ml syringe equipped with a 25G needle. To collect the total membrane fraction, the suspension was centrifuged for 30 min at 336,896 g (Beckmann MLA80 rotor) to remove the proteins of the mitochondrial intermembrane space and matrix. The pellet, which contains outer and inner membranes, was resuspended in up to 500 μl of 8.5% (w/v) of sucrose dissolved in 10 mM Tris-HCI (pH 7.4) and applied on a discontinuous sucrose density gradient [1.5 ml of 25%, 38%, 52%, and 61.5% of sucrose (w/v) in 10 mM Tris-HCI (pH 7.4) at a final volume of 1.5 ml each] and centrifuged for 12 min at 170,000 g (Beckmann MLA80 rotor). The outer membranes are recovered from the top sucrose layer fractions (between 8.5% and 25% w/v) and IMMs are recovered from the boundary of sucrose layer fractions 38% and 52% (w/v). The fractions containing the OMM and the IMM were eventually diluted with 10 mM Tris-HCl (pH 7.4) until fractions contain ∼15% of sucrose and centrifuged for 30 min at 336,896 g (Beckmann MLA80 rotor). The obtained pellets of the OMM and IMM were resuspended in 100 μl of 10 mM Tris-HCI (pH 7.4), flash frozen and stored until use in −80°C.

### Isolation of OMM and IMM lipid extracts

Lipid extraction of the isolated OMM and IMM membranes was performed according to Bligh and Dyer (Bligh and Dyer, 1959), where 0.8 volume of membranes at a total protein concentration of 1 mg/ml (OMM or IMM) were mixed with one volume of chloroform and two volumes of methanol [chloroform/methanol/water mixture (1:2:0.8; v/v/v)]. After thoroughly vortexing, one additional volume of chloroform is added to the suspension and again thoroughly vortexed. Next, one volume of distilled water is added [chloroform/methanol/water mixture at (2:2:1.8; v/v/v)] and after mixing the suspension incubated for 30 min at room temperature. To completely phase separate the aqueous from the organic phase, the suspension is centrifuged for 10 min at 12,000 × g (Beckmann F301.5 rotor) at 4°C and recovered the lower organic phase containing the lipids and dried under a stream of nitrogen and stored until further use.

### Determination of lipid concentration of OMM and IMM lipid extracts

The dried extracted lipid extracts were resuspended in chloroform and the lipid concentration was estimated according to Fiske and Subbarow (King, 1932). 10 μl of lipid extract was pipetted in triplicate into glass tubes and dried at 200°C–215°C. Then the samples were digested for 25 min at 200°C–215°C in the presence of 225 μl of 9N H_2_SO_4_. During all incubation steps, except for the first drying one, the open glass tubes were sealed with a glass marble to prevent evaporation. After cooling to RT, the samples were supplemented with 75 μl of commercial H_2_O_2_ and incubated at 200–215°C for additional 30 min to remove any present coloration of the samples. For the colorimetric assay, the colourless samples were cooled down to RT and first supplemented with 1.95 ml of milliQ water and 250 μl of 2.5% (w/v) of ammonium molybdate (VI) tetrahydrate. After thoroughly vortexing, the samples were supplemented sequentially with 250 μl of 10% (w/v) of ascorbic acid and thoroughly vortexed. The samples were then incubated for 7 min at 100°C and after cooling, 200 μl of each sample was transferred to a 96-well plate to read the absorption at 750 nm with a GEMINI XS fluorescence plate reader (Spectramax). To obtain a lipid concentration, the measured absorption of the samples was then referred to the absorption of standard curve ranging from 0 to 0.228 μmoles of KH_2_PO_4_.

### Lipid identification and analysis

To compare the qualitative profile of inner and outer mitochondrial membranes, a single mono-dimensional thin layer chromatography (TLC) plate was prepared using a combination of solvents able of showing as many of the membrane lipid classes as possible. 10 μg was taken from the OMM and IMM extract and spotted onto a high performance-TLC silica gel plate (HP−TLC; Merck). The plate was first run with chloroform/methanol/acetic acid/water (50:37.5:3.5:2; v/v/v/v) up to the middle of the plates to resolve the phospholipid classes. Then, to concentrate the neutral lipids into a single sharp band, after drying the plate, diethyl ether was run until the solvent front was passed by 1 cm to finally run hexane/ether (80:20; v/v) up to the top of the plates to resolve the neutral lipids present in the sample. Lipid standards have been employed simultaneously to identify each spot of the OMM and IMM extracts. After running the TLC, lipid spots were assessed by charring-densitometry. The plates were dried and dipped into an aqueous solution of 3% (w/v) cupric acetate and 8% (v/v) phosphoric acid. Then, the plates were allowed to dry for 10 min at room temperature and heated for 10–15 min at 180°C to carbonize the lipids (Entezami et al., 1987). The semi-quantification of lipid stains was performed with the ImageJ software package (NIH) (Schneider et al., 2012) and the percentage of each lipid spot was calculated relative to the sum of all spots. Note that the percentage of cholesterol was estimated using the same densitometry analysis. As the total intensity of each spot is proportional to amount of material in each lipid spot, and assuming a similar density for all lipid species, the percentages given in our estimates correspond to % w.

### Surface tension adsorption isotherms

The equilibrium surface tension of aqueous solutions of OMM and IMM lipid extracts at different phosphorous concentrations was measured at 22°C using a paper Wilhelmy plate. The standard deviation was ±0.2 mN/m.

### Compression isotherms

Compression isotherms were obtained using a computer controlled Langmuir balance (611 model from NIMA, UK). The surface pressure was measured using a paper Wilhelmy plate. In order to prevent for surface perturbations induced by the air convection streams and to avoid undesirable dust contamination, the whole set-up is covered by a transparent Plexiglas case. Before each measurement, the subphase surface is cleaned by sweeping and suction. Small aliquots (10 μl typically) of the chloroform lipid solutions were spread on the aqueous surface with a Hamilton syringe. All experiments were carried out at 22°C. Compression isotherms were recorded upon compression of a diluted monolayer at a constant compression rate of 10 cm^2^/min. The surface pressure is plotted against the mean molecular area, as obtained by dividing the total surface area by the numerical amount of phosphorous spread on the surface.

### Supported lipid bilayers

The OMM and the IMM lipid extracts were fluorescently labelled with Rhodamine-PE (RhPE; 0.1% mol) and dried with a speedvac concentrator at room temperature. The film was resuspended in phosphorous buffered saline (PBS) to lipid final concentration of 1 mg/ml. The lipid suspension was sonicated with a tip sonicator (Vibra-Cell 75115 Ultrasonic Liquid Processor) for 30 min in 5 min on-off cycles (40% power) to form unilamellar lipsomes. Supported lipid bilayers (SLBs) were prepared in custom-made observation chambers (200 μl) on a thoroughly cleaned coverslip (Menzel, 60 mm × 60 mm). Prior to liposome spreading, coverslips were incubated with 100 μl of 10% (w/v) methoxysilane (3-[methoxy (polyethyleneoxy)9-12]-propyltrimethoxysilane, Geles) for 30 min and at room temperature to improve the wettability of the glass surface (Halliwell and Cass, 2001). After removing the excess silane, the reaction chamber was carefully washed with PBS and liposomes (60 μM final concentration) were incubated for 30 min in PBS supplemented with CaCl_2_ (20 mM final concentration). Finally, SLBs were washed with PBS to remove CaCl_2_ and non-spread lipid material. For microscopy, the observation chamber was finally filled with 200 μl of PBS.

### Confocal laser scanning microscopy

Confocal images were acquired using a Nikon Ti-E inverted microscope equipped with a Nikon C2 scanning confocal module, a Nikon Plan Apo 100X NA 1.45 oil immersion objective and filter cubes. Images were captured with Nikon NIS-Elements software package.

### Fluorescence recovery after photobleaching

Standard Fluorescence recovery after photobleaching (FRAP) measurements on SLBs were performed with a Nikon Ti-E inverted microscope as described for CLSM. Random zones on SLBs were photobleached and the recovery of fluorescence intensity at the bleached zone was monitored and corrected for image alignment and photobleaching with ImageJ software package. The fluorescence recovery curves were fitted to (Yguerabide et al., 1982):

$$f\left(t\right)=\frac{\;f\left(0\right)+f\left(\infty \right)\cdot \left(t/t_{1/2}\right)}{1+\left(t/t_{1/2}\right)}$$ (1)

where f(t) is the time normalized fluorescence intensity ${F\left(t\right)/F}_{o}$ , $F_{o}$ is the fluorescence intensity just before photobleaching, f(0) is the normalized fluorescence intensity just after photobleaching, f(∞) is the normalized maximum fluorescence intensity and $t_{1/2}$ is the half-time of the fluorescence recovery. As $t_{1/2}$ depends on the percent bleach of the sample, the diffusion coefficient, D, was then calculated applying the correction factor β as follows (Axelrod et al., 1976).

$$D=\frac{w^{2}}{{4\cdot t}_{1/2}}\beta$$ (2)

where β accounts for both the beam shape and the bleaching extent (Yguerabide et al., 1982) and w is the bleached radius.

### Formation of giant unilamellar vesicles

Giant vesicles were prepared using the standard electroformation protocol (Mathivet et al., 1996). The fabrication chamber was composed of two 1-mm spaced conductor indium tin oxide (ITO)-coated slides (7.5 cm^2^ × 2.5 cm^2^; 15–25 Ω/sq surface resistivity; Sigma). Briefly, giant unilamellar vesicles (GUVs) were prepared by transferring a volume of 15–20 μl of purified IMM or OMM in chloroform (1 mM phosphorous) onto each ITO slide. Samples are dried at room temperature and rehydrated in sucrose solution (300 mM) and the electrodes were connected to an AC power supply (10 Hz, 1.1 V; Agilent) for 2 h at room temperature and 50°C for IMM and OMM lipid extracts, respectively. For fluorescence microscopy, OMM and IMM lipids were supplemented with 0.2% mol RhPE.

### Micropipette aspiration experiments

To measure the bending modulus with the micromanipulation device, a micrometer-sized GUV (20 μm typically) is pulled out by a cylindrical micropipette in the aspiration mode (Longo and Ly, 2007). In a suction experiment, the pressure difference Δp between the vesicle interior and the pipette determines the membrane tension, σ , as $\sigma =\Delta p\frac{R_{p}}{2}\left(1-\frac{R_{p}}{R_{V}}\right)$ ; where $R_{p}$ is the micropipette radius and $R_{V}$ the vesicle radius. For a vesicle under suction at a given Δp , a protrusion length, $L_{p}$ , is aspirated inside the pipette and the relative excess area $\alpha =\frac{A-A_{0}}{A_{0}}=\frac{\Delta A}{A_{0}}$ is given by $\alpha =\frac{\Delta L_{p}}{2R_{p}}\left[{\left(\frac{R_{p}}{R_{V}}\right)}^{2}-{\left(\frac{R_{p}}{R_{V}}\right)}^{3}\right]$ ; where $\Delta L_{p}$ is the variation of the protrusion length from an increase in suction pressure compared to the protrusion length measured at an initial low tension state, $\sigma_{0}$ . For low tensions, the entropic regime dominates (Evans and Rawicz, 1990) and the bending modulus is obtained from the linear fitting of the Canham-Helfrich equation which connects the relative excess area α and the membrane tension σ through (Evans and Rawicz, 1990):

$$\ln\left(\frac{\sigma }{\sigma_{0}}\right)\approx \frac{8\pi \kappa \alpha }{k_{B}T}$$ (3)

where κ is the bending modulus of the vesicle and $k_{B}T$ is the thermal energy.

## Results

### Isolation of mitochondrial lipids

**FIGURE 1:**
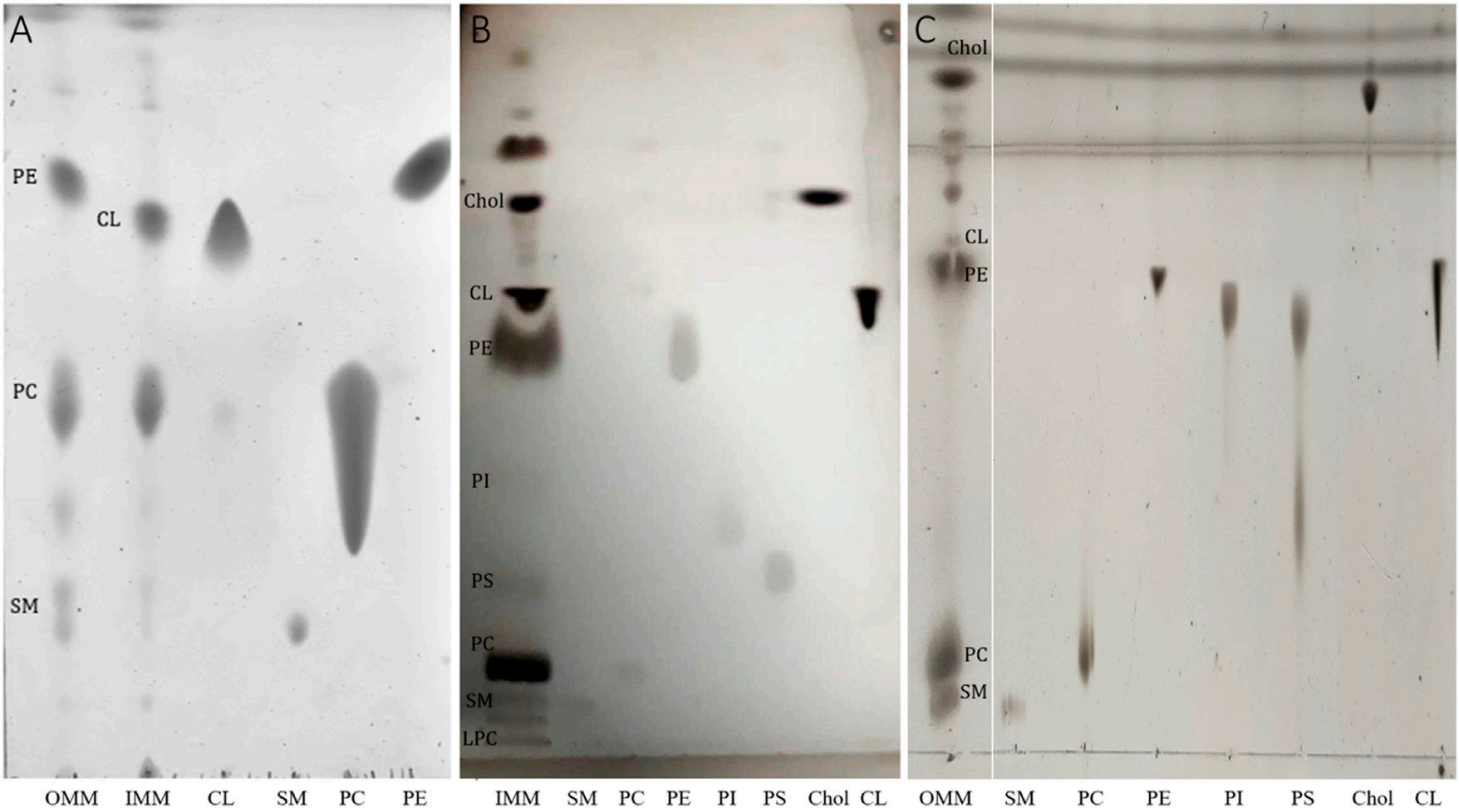
**(A)** Representative TLC showing lipid profiles of OMM and IMM lipid extracts. TLC was prepared by spotting aliquots taken from membrane lipid extracts containing a fixed amount (5 μg) of total lipid phosphorus. The long tail of the PC standards is observed at high lipid concentration. **(B)** Representative TLC showing the lipid profile of IMM using three different running solvents (see methods for details). **(C)** Representative TLC showing the lipid profile of OMM using three different running solvents. The lipid abbreviations correspond to: Chol, cholesterol; CL, cardiolipin; PE, phosphatidylethanolamine, PS, phosphatidylserine; PI, phosphatidylinositol; phosphatidylcholine; SM, sphingomyelin.

Mitochondrial membranes were extracted from pig heart mitochondria by density gradient centrifugation after mechanical breakdown (Smith, 1967). Then, the lipids were extracted from the isolated OMM and IMM through liquid-liquid separation (Bligh and Dyer, 1959). To characterize the extracted lipid mixtures and to check for their lipid compositions we performed standard mono-dimensional thin layer chromatography (TLC), run with different solvents to specifically separate the polar from the non-polar lipids as described in experimental procedures. To identify the individual lipid bands we have simultaneously spotted a series of lipid standards representing the expected lipids present in OMM and IMM (Horvath and Daum, 2013). We can clearly distinguish a difference in lipid pattern for the OMM and IMM (Figure 1A). As expected, the OMM lipid extract is mainly composed of phosphatidylcholine (PC), phosphatidylethanolamine (PE), and sphingomyelin (SM), whereas the IMM contains cardiolipin (CL). Interestingly, bacterial cell membranes and the inner mitochondrial membrane have a relatively high proportion of CL—between 10% and 20% of the total lipid content of these membranes is made up of CL (Horvath and Daum, 2013), a lipid that is practically absent from other biological membranes (van Meer et al., 2008). However, no PE was detected in the IMM fraction. As PE and CL are known to run similarly in more polar solvents, a multiple-step TLC was run with three different solvents (see methods) and the PE and CL spots were clearly resolved in the IMM fraction (Figure 1B). The semi-quantification of lipid stains from three independent experiments indicated an enrichment of cholesterol, CL, PE and PC in the IMM fraction (10%, 21%, 34%, and 27%, respectively). Additionally, minor components were also detected: lysophosphatidylcholine (LPC), sphingomyelin (SM), phosphatidylserine (PS), and phosphatidylinositol (PI) (0.7%, 1.6%, 2.4%, 2.2% of the total phospholipid content, respectively). As for OMM (Figure 1C), the multiple-step TLC indicated an enrichment of PC, PE, SM, and cholesterol (35%, 41%, 6.3%, and 16.7%, respectively). The total lipid composition of OMM was completed with CL (1%). Although a complete analysis of fatty-acyl chain profile was not performed, the absence (1%) and the presence (up to 21%) of CL in the OMM and IMM fractions, respectively, were taken as an indication of a clean separation (See Table 1 for a comparative lipid composition between IMM and OMM).

**TABLE 1:** Lipid composition of the outer (OMM) and inner (IMM) lipid extracts from porcine heart.

	OMM	IMM	OMM	IMM	OMM	IMM
	(% of total phospholipids, including cholesterol)		(% of total phospholipids, excluding cholesterol)		(% of total phospholipids)^a^	
Phosphatidylcholine (PC)	35.0 ± 3.0	27.4 ± 2.0	42.0 ± 3.0	30.5 ± 2.0	56.3	26.5
Phosphatidylethanolamine (PE)	41.0 ± 3.0	34.4 ± 3.0	49.2 ± 3.0	38.3 ± 3.0	28	37.9
Cardiolipin (CL)^b^	1.0 ± 0.2	21.0 ± 2.0	1.2 ± 0.3	23.4 ± 2.0	0.4	25.4
Sphingomyelin (SM)	6.3 ± 0.3	1.6 ± 0.1	7.6 ± 0.5	1.8 ± 0.1	4.5	0.5
Phosphatidylinositol (PI)	—	2.2 ± 0.3	—	2.5 ± 0.3	9.3	3.4
Phophatidyl serine (PS)	—	2.4 ± 0.5	—	2.7 ± 0.5	0	0
Cholesterol (Chol)	16.7 ± 2.0	10.3 ± 1.0	—	—	c	c
Lysophosphatidylcholine	—	0.7 ± 0.2	—	0.8 ± 0.1	1.3	0.6
Lysophosphatidylethanolamine	—	—	—	—	0	1.3

a From reference (Comte et al., 1976) as expressed as percentages of total lipid phosphorous.

b Including phosphatidic acid.

c Unlike our work, the cholesterol content was quantified separately using a standard colorimetric assay based on the Zlatkis-Zak reaction. Values represent mean ± SEM of three independent experiments.

### Adsorption properties

**FIGURE 2:**
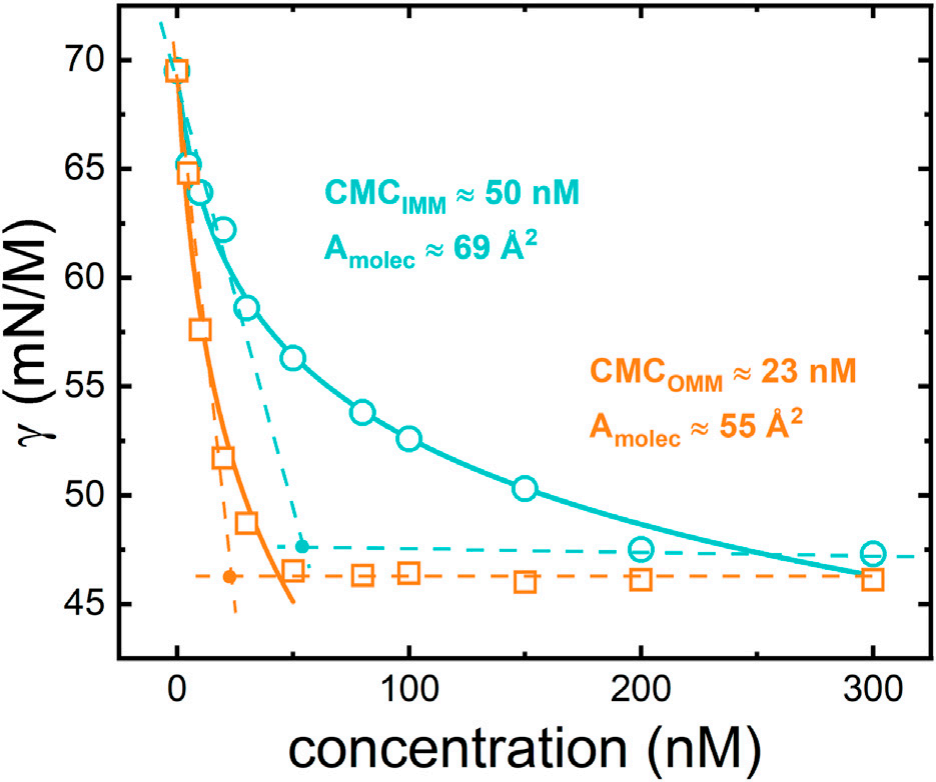
Equilibrium adsorption isotherms for IMM (circles) and OMM (squares) lipid extracts obtained from porcine heart. The experimental points are fitted using the Langmuir-Gibbs equation (see Eq. 4 and text for details).

The Gibbs adsorption isotherms at 20°C of OMM and IMM lipid extracts at the air-water interface were determined by measuring the surface tension of aqueous solutions with different lipid concentrations (Figure 2). At low concentration and for both OMM and IMM lipid extracts, the surface tension monotonically decreased as the bulk concentration increased, corresponding to the monomeric adsorption of lipids to the interface. At higher concentrations, the surface tension reached a plateau value, which was similar for both lipid extracts $\left(\gamma_{\min}\approx 46\;mN/m\right)$ . However, the minimal surface tension was reached progressively for IMM whereas the OMM lipid extract behaved as an insoluble surfactant, i.e., once the minimal value of surface tension was reached the surface concentration was no longer modified. The bulk concentration leading to this transition provides the critical micelle concentration or CMC, where the bulk surfactants aggregate into micelles. The CMC was found to be 23 nM for OMM and 50 nM for the aqueous IMM lipid solution (Figure 2). Assuming that 1) the activity coefficient of both OMM and IMM extracts are close to unity for the studied concentration range and 2) the lipid mixture behaves as a single and non-ionic surfactant; the variation of the surface tension, γ , with the bulk lipid concentration could be fitted to the Gibbs-Langmuir equation of state:

$$\gamma =\gamma_{0}-RT\Gamma_{\infty }\ln\left(1+Kc\right)$$ (4)

where $\gamma_{0}$ is the surface tension of the bare air/water interface, $\Gamma_{\infty }$ is the maximum surface concentration, K is the adsorption equilibrium constant, c is the lipid concentration, R is the gas constant and T is the absolute temperature. From the fitting curves, we obtained the adsorption equilibrium constant K , $K_{OMM}=0.42\;{\pm \;0.3\;nM}^{-1}$ and $K_{IMM}=0.15\;\pm 0.04\;nM^{-1}$ . Also, the maximum surface concentration was obtained from the fit, with $\Gamma_{\infty }\approx {\left(3.0\pm \;0.7\right)\times 10}^{-6}$ and ${\left(2.4\;\pm \;0.3\right)\times 10}^{-6}\;mol/m^{2}$ for OMM and IMM lipids, respectively. The mean molecular area can be calculated through A=1Γ∞NA , with A≈55 and 69 ${Å}^{2}$ for OMM and IMM lipids, respectively. As the mean molecular area is expected to be inversely related to the average lipid packing within the monolayer, higher values of A are indicative for more expanded membranes, so that OMM lipids exhibit stronger intermolecular interactions than IMM lipids.

### Compression isotherms

**FIGURE 3:**
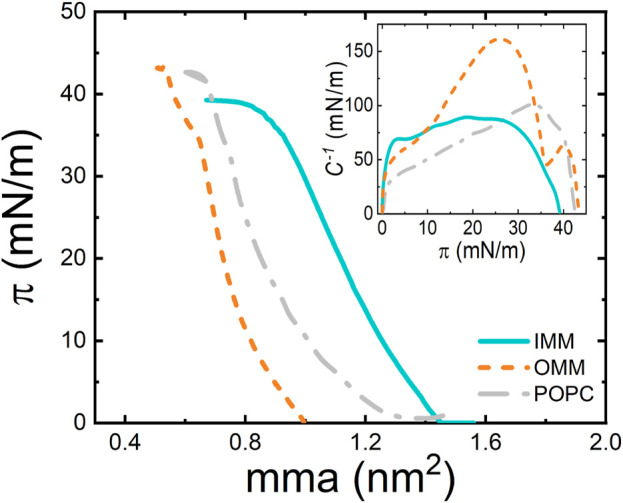
π−A isotherm of POPC, OMM, and IMM lipid monolayers at 22°C and at compression rate of 10 cm^2^/min. (Inset) Compressibility modulus, $C^{-1}$ , of POPC, OMM, and IMM lipid extracts as a function of the surface pressure.

The physical state of a monolayer can be also studied by monitoring its surface pressure upon compression at a particular temperature. Compression approaches the molecules to each other promoting transitions from different phases, which are characterized by particular orientation and packing of molecules on the interface. The experimental pressure-area (π-A) compression isotherms of OMM and IMM lipid extracts were obtained in a Langmuir trough at T = 25°C (Figure 3). Remarkably, IMM lipids exhibited larger areas per molecule than OMM lipids, i.e., OMM behaved as a more packed monolayer compatible with the presence of cholesterol in the lipid composition (Figure 1). At lower compression, the surface pressure remained close to 0 mN/m below ≈90 and $\approx 140\;{Å}^{2}$ for OMM and IMM, respectively. At further compression, the lateral pressure of IMM monolayers increased with a monotonic expanded-like behaviour without phase transition plateaux. Then, IMM reached the collapse regime at a mean molecular area $A\approx 80\;{Å}^{2}$ , characterized by a constant pressure $\pi_{c}\approx 40\;mN/m$ . The compression isotherm obtained for IMM is similar to those measured for POPC (see Figure 3) or *E. coli* lipids (Lopez-Montero et al., 2008), (Lopez-Montero et al., 2010). Unlike IMM or POPC, the expanded regime of OMM monolayers transitioned to a condensed-like phase at an area close to $65\;{Å}^{2}$ and at surface pressures typical of the bilayer packing state (Marsh, 1996). The π-A monolayer profile at the condensed-like phase was characterized by a higher slope and entered the collapse regime at a mean molecular area of $\approx 55\;{Å}^{2}$ . The monolayer collapse pressure of OMM was higher than IMM and achieved at $\pi_{c}\approx 43\;mN/m$ .

A useful parameter for lipid monolayer characterization is the compression modulus, $C^{-1}$ , which takes higher values for more condensed monolayers. The equilibrium compressibility modulus (Figure 3, inset), as defined by the change in monolayer pressure caused by an infinitesimal change in the molecular area, can be calculated from the numerical derivative of the experimental π-A isotherms (see Methods). Similar to POPC and *E. coli* monolayers, the compressibility modulus of IMM lipids displays a maximum ( $C^{-1}\approx 80\;mN/m$ ) at the bilayer lipid packing state (30–35 mN/m) and drops to zero when reaching the collapse pressure. For OMM lipids, the monolayer was highly compressible ( $C^{-1}\approx 50\;mN/m$ ) in the more expanded-like regime ( π<10 mN/m ) but the compressibility modulus increased to a maximum value ( $C^{-1}\approx 150\;mN/m$ ) just before reaching the pseudoplateau ( π≈35 mN/m ). This value is compatible with compacted monolayers (Wydro, 2012). Again, the maximal rigidity appeared at surface pressures corresponding to the biologically relevant surface packing (Marsh, 1996) and further compression reduced $C^{-1}$ down to smaller values compatible with the collapsed arrangement.

### Diffusive properties in supported lipid bilayers

**FIGURE 4:**
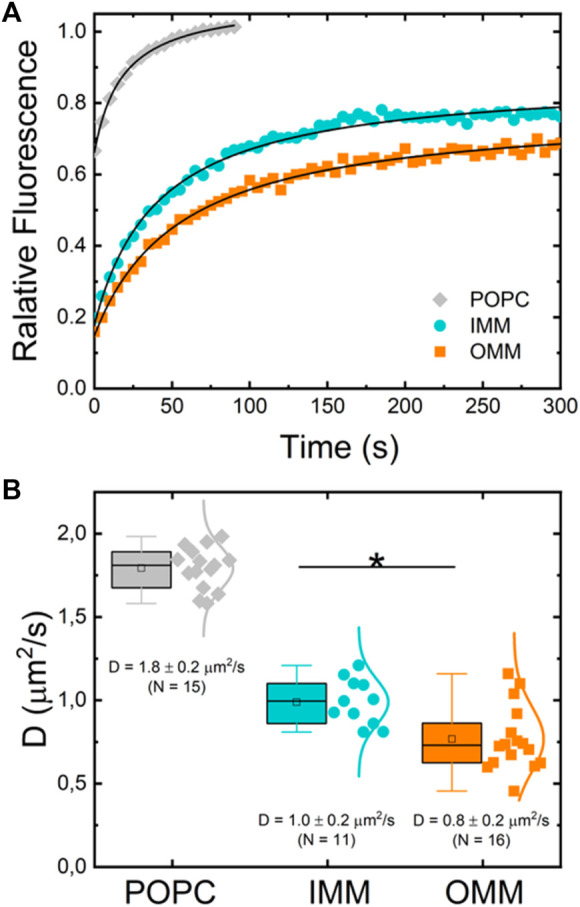
**(A)** Typical normalized fluorescence recovery curves of supported lipid bilayers of POPC (gray diamonds), IMM lipid extract (cyan circles) and OMM lipid extract (orange squares) supplemented with 0.1% mol of fluorescent lipid RhPE. **(B)** Diffusion coefficient of RhPE in POPC (*N* = 15), OMM (*N* = 16), and IMM (*N* = 11) lipid bilayers obtained from FRAP curves.

To further characterize the dynamic properties of both lipid extracts we investigated the effect of the lipid composition on the lateral mobility of a fluorescently labelled lipid (RhPE) embedded in lipid membranes built as supported bilayers (SLBs). This configuration allows for a quantitative measurement of the diffusion coefficients of fluorescent probes through Fluorescence Recovery After Photobleaching (FRAP). A zone in the lipid bilayers was selectively photobleached by a high laser power scanning. The recovery of fluorescence intensity at the bleached zone occurs through Brownian diffusion of surrounding unbleached probes (Figure 4A). The measurement of a single characteristic diffusion time enables to obtain the diffusion coefficient of the probe in the embedding bilayer (see Methods) (Axelrod et al., 1976; Yguerabide et al., 1982). Figure 4A shows typical FRAP curves for RhPE embedded in SLBs made of POPC, OMM, and IMM lipid extracts. The labelled phospholipid RhPE followed unrestricted Brownian motion with diffusion coefficients $D_{POPC}=\left(1.8\pm \;0.2\right)\;{\mu m}^{2}/s$ , $D_{OMM}=\left(0.8\;\pm \;0.2\right)\;{\mu m}^{2}/s$ and $D_{IMM}=\left(1.0\pm \;0.2\right)\;{\mu m}^{2}/s$ when embedded in POPC, OMM and IMM lipid bilayers, respectively (Figure 4B). The diffusion coefficient measured for RhPE in mitochondrial lipid bilayers was in agreement with previous studies using FRAP (Merzlyakov et al., 2006) or FCS experiments (Bockmann et al., 2003). However, the immobile fraction was higher (up to 30%) for lipid extracts than for POPC (<10%) The presence of lipid heterogeneities and defects in SLBs made of mitochondrial lipid extracts might explain the high immobile fraction.

### Formation of giant unilamellar vesicles and phase immiscibility properties

**FIGURE 5:**
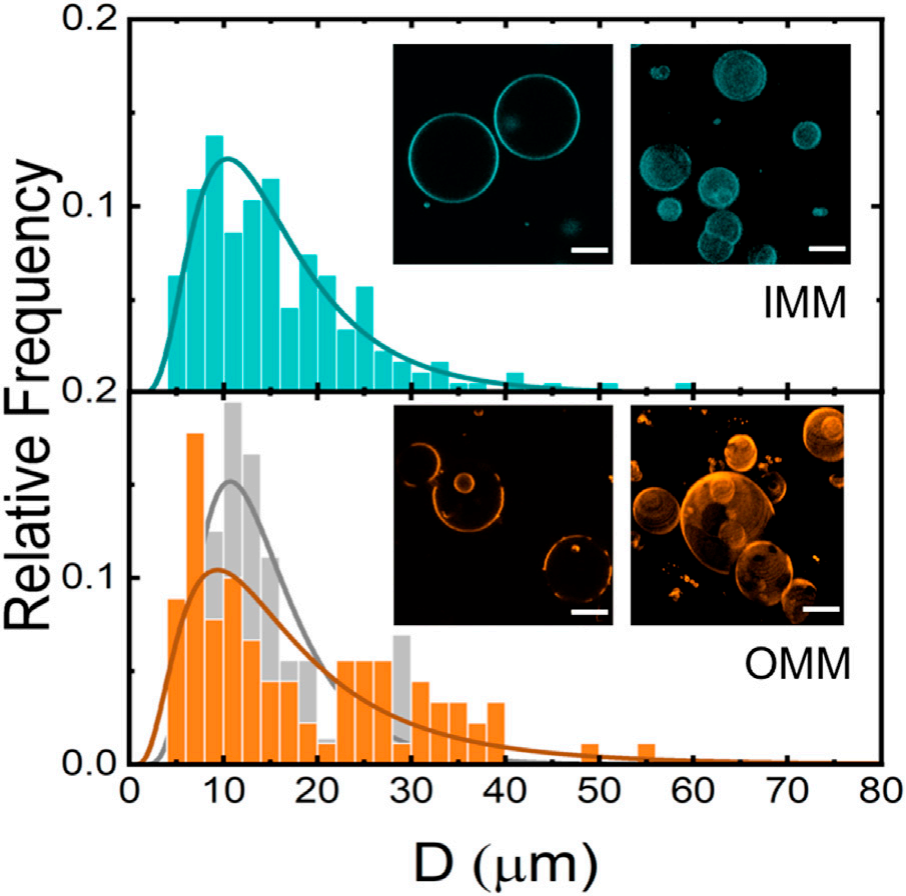
Size distribution of IMM- (*N* = 312, cyan) and OMM- (*N* = 261, orange) and POPC-GUVs (*N* = 237, gray). Insets: Confocal fluorescence microscopy images of IMM and OMM-GUVs. Unstained areas correspond to *l*_*o*_ domains. Scale bars are 10 μm.

Using a classic electroformation protocol (Mathivet et al., 1996) we succeeded in preparing GUVs from mitochondrial lipid extracts (Figure 5). Standard settings (0.5 V/mm and 10 Hz) were used for GUV electroformation. However, the electroformation chamber and the sucrose solution was kept at 50°C during GUV formation for the OMM lipid extract. The high experimental temperature is compatible with an OMM lipid composition enriched with cholesterol and/or high-melting temperature phospholipids, such as SM (see Figure 1C; Table 1). Fluid lipid membranes are required for GUV fabrication to favour lipid mixing during electroformation. We named the vesicles IMM-GUVs and OMM-GUVs for GUVs made of inner and outer mitochondrial lipids, respectively. Both OMM- and IMM-GUVs were almost spherical with a variable size ranging from 5 to 60 μm . Unlike IMM-GUVs (2%), 90% of OMM-GUVs exhibited micron-scale liquid immiscibility (Veatch and Keller, 2003) as visualized by the selective partition of RhPE into liquid disordered (*l*_*d*_) phases. In contrast, coexisting liquid ordered (*l*_*o*_) phases excluded the fluorescent probe and were imaged as dark membrane regions (Figure 5, inset). To further characterize IMM- and OMM-GUVs, the size distribution of a population of giant vesicles was determined. For that, several *z*-stacks of the different observation fields of sedimented vesicles were taken. For each vesicle, the true diameter is assumed when the size of the vesicle reached its maximal size under confocal observation. The size distribution of GUVs was represented in histograms obtained from a population of tens of vesicles showing a large population of IMM-GUVs and OMM-GUVs with a typical size above 10 μm . The histograms displayed a similar size distribution obtained for simple GUV model made of POPC (Figure 5).

### Bending moduli obtained from micropipettes

**FIGURE 6:**
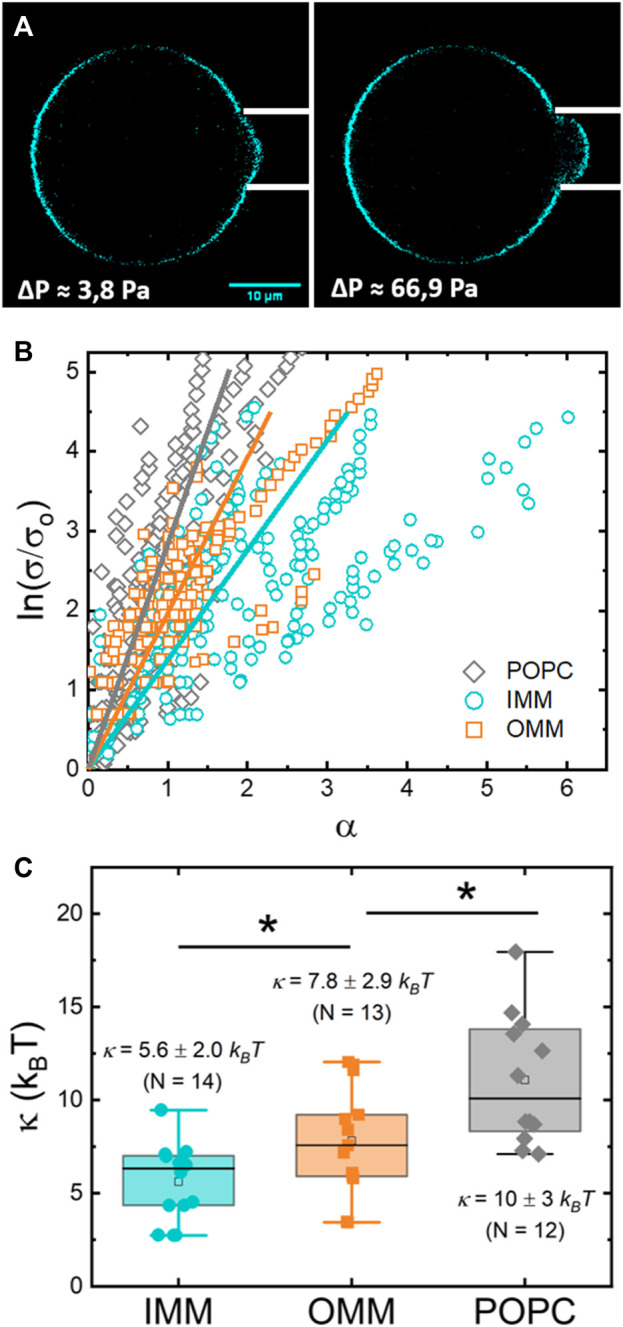
**(A)** Confocal micrograph of a IMM-GUV aspirated in a micropipette at low (left) and medium (right) tension. The change in the length of the protrusion is proportional to the change in the relative excess area α . **(B)** Plot of the logarithm of the relative tension ( $\sigma /\sigma_{0}$ ) versus the relative excess area α for different POPC-, IMM-, and OMM-GUVs. **(C)** Box plots comparing the bending moduli for POPC-, IMM-, and OMM- GUVs (*N* = 12, *N* = 14, and *N* = 13, respectively). The median is represented with a line; the box represents the 25th–75th percentiles; and error bars show the 5th–95th percentile. Statistical significance: * (*p* ≤ 0.05).

GUVs are membrane models that allow their mechanical characterization under external forces. The mechanical parameters define the compliance of the membrane to be shaped when exposed to different modes of deformation. In particular, the bending modulus provides the basic energy scale for bending deformations. The bending modulus of OMM- and IMM-GUVs was measured by micropipette aspiration (Evans and Rawicz, 1990) (see Methods and Figure 6A) from the fitting of Eq. 3 to the experimental data (Figure 6B).

A first set of experiments provided the bending modulus of IMM lipids (Figure 6C), with $\kappa_{IMM}=5.6\pm \;2.0\;k_{B}T$ , (*N* = 14). This value agrees with previously reported measurements for cardiolipin-containing GUVs made of *E. coli* lipid extracts and measured with flickering spectroscopy (Almendro-Vedia et al., 2017). For OMM-GUVs, the absolute values of bending moduli for the *l*_*d*_ and *l*_*o*_ phases cannot be obtained from phase-separated vesicles (Tian et al., 2007). For simplicity, we performed the experiments on non phase-separated OMM-GUVs, obtaining a higher bending modulus of $\kappa_{OMM}=7.8\pm \;2.9\;k_{B}T$ , (*N* = 13). This value, although underestimated, is in agreement with previously reported measurements for cholesterol- and SM-containing fluid membranes (Gracia et al., 2010). For comparison, POPC GUVs were characterized by a bending modulus of $\kappa_{POPC}=10\pm 3\;k_{B}T$ in agreement with (Niggemann et al., 1995).

## Discussion

Cell membranes are composed of a heterogenous mixture of lipids, which are evenly distributed across the cell and the membranes themselves. The lipid composition plays different roles in membrane dynamics and often regulates protein function in fundamental processes such as cell division, cell signalling or apoptosis (van Meer et al., 2008). To better understand membrane functions, it is crucial to understand how lipids interact with each other and with other membrane components. To study lipid–lipid interactions, membrane models such as monolayers, vesicles, and planar bilayers are extensively used as they can be studied with multiple biophysical techniques (Rosilio, 2018).

Artificial membrane models allow the control of the lipid composition varying from a single saturated or unsaturated lipid to complex lipid–mixtures that are enriched in particular lipid species to mimic different biomembranes. For example, PG is generally chosen to mimic the negatively charged lipids found in bacterial membranes (Clausell et al., 2007) and asymmetric lipopolysaccharide-phospholipid bilayers are built to mimic the outer membrane of Gram-negative bacteria (Michel et al., 2017). The extensively studied DPPC represents the paradigmatic lipid to model the lung surfactant (Perez-Gil, 2022). Microphase separated vesicles composed of PC/SM/cholesterol are proposed to mimic raft-like domains with coexisting liquid ordered (*l*_*o*_) and liquid disordered (*l*_*d*_) phases (Veatch and Keller, 2003). PS-enriched membranes model the endoplasmic reticulum (Lopes et al., 2014) whereas the non-bilayer PE is included in fusogenic-mimicking membranes due to its ability to form inverted hexagonal phases, which facilitates protein-independent membrane destabilization and fusion (Rappolt et al., 2003). SM and ceramide-enriched membranes are known to model apoptotic membranes (Catapano et al., 2011; Lopez-Montero et al., 2013; Catapano et al., 2015; Catapano et al., 2017). Also, the inner mitochondrial membrane is often mimicked by including CL to the lipid formulation (Domenech et al., 2006). Although the comprehension of lipid-lipid interactions requires the use of simple models; they seem too simple to mimic the real complexity intrinsic to living systems. Then, native membranes and lipid extracts appear to be a more suitable alternative.

Unlike bacterial lipid extracts, eukaryotic lipid extracts are not commercially available, mainly due to the lack of reliable and simple protocols to isolate the different biomembranes without significant cross-contaminations or to obtain detergent-free samples. This applies specially to mitochondrial membranes, where the outer membrane has to be physically separated from the inner membrane. The existence of multiple membrane contacts between OMM and IMM through protein complexes (Vyssokikh and Brdiczka, 2003) makes the separating procedure a challenging task. OMM and IMM can be separated by incubating mitochondria in hypotonic conditions that provoke the swelling of mitochondria (Smith, 1967). This simple protocol is relatively efficient when dealing with tissue mitochondria (liver, brain, or heart) but difficult to perform with mitochondria from cultured cells as they do not swell, even in the presence of particular antibiotics such as alamethicin (Gostimskaya et al., 2003). This channel-forming peptide binds strongly to lipid bilayers and forms voltage-dependent channels (Vodyanoy et al., 1983), which results in mitochondrial swelling (Brustovetsky et al., 2002).

In our case, a first indication of the limited ability of mitochondria to swell was the dissimilar isolation yield obtained for OMM and IMM after treatment. Whereas a visible pellet was obtained for IMM, a reduced precipitate was seen for OMM after differential centrifugation and sucrose density gradients. As a result, a more than ten-fold IMM/OMM ratio was often found after phosphorous determination of the lipid extracts. Table 1 contains the lipid composition of both OMM and IMM lipid extracts, including cholesterol, assessed by charring-densitometry. As expected, a similar lipid composition to previous analysis of porcine heart lipid extracts was found (Comte et al., 1976). Mainly, IMM was enriched in PE, PC, and CL whereas OMM was enriched in PE, PC, and SM. However, we found here that both OMM and IMM lipid extracts contained an unusual high levels of cholesterol (>10%). As for other mitochondria from other tissues or species (Horvath and Daum, 2013), PC and PE were also the major mitochondrial phospholipid classes. Moreover, the IMM was enriched with CL, a mitochondrial hallmark found in all organisms, including plants (Daum and Vance, 1997). Among the dissimilarities with precedent reports on lipidomics of mitochondria, it is noteworthy the small amount of PI present in the OMM as compared to other mammalian cells or plants (Daum and Vance, 1997). Instead, SM was present in large quantity in the OMM, a phospholipid relatively scarce in rat liver mitochondria (Daum and Vance, 1997). Also, the cholesterol levels in rat liver mitochondria are high in the OMM, reaching up to 10% of the total lipid content, whereas nearly absent in the IMM (Daum and Vance, 1997). The high levels of cholesterol found here are still lower than other subcellular compartments.

As TLC is limited to the identification of lipid species, a further characterization to determine the number of fatty acyl carbons, the number of fatty acyl double bonds and its position across the acyl chain is required for a complete characterization of the OMM and IMM lipid extracts. The complete lipidome of an organelle needs the combination of various methods, most of them based on liquid chromatography-mass spectrometry (LC-MS) (Kofeler et al., 2012). In our particular case, the detection and quantification of plasmenyl acyl chains would be indicative for a good extraction, as both OMM and IMM are enriched in plasmenyl-PC and plasmenyl-PE, respectively (Comte et al., 1976). Our main goal was, however, to report precise values of the viscoelastic parameters to mechanically characterize both the OMM and IMM lipid extracts and determine if a different interfacial behaviour is sustained by the lipid complexity after extraction and purification. The absence of CL in OMM lipid samples as detected in TLC plates was adopted as a quality control for a good separation of OMM and IMM membranes and to further characterize the lipid extracts. This criterion allowed us to clearly allocate the different physicochemical properties of the lipid extracts to their different lipid composition*.* Overall, OMM lipid extract was enriched in PE, PC, SM, and cholesterol and IMM lipids mostly contained PE, PC, CL, and cholesterol (Figure 1). As a result, we found that OMM lipids form more compact and stiffer membranes than IMM membranes, which are in turn characterized by a higher mean molecular area and lower bending and compression moduli (Table 2).

**TABLE 2:** Physicochemical parameters of OMM and IMM lipid extracts.

	A_Gibbs_ ( ${Å}^{2}$ )	CMC (nM)	A_collapse_ ( ${Å}^{2}$ )	*C* ^−1^ (mN/m)^b^	*D*_*0*_ (μm^2^/s)	*κ* (k_B_T)	Phase separation
OMM	55	23	55	150	0.8 ± 0.2	8 ± 3	Yes
IMM	69	50	80^a^	80	1.1 ± 0.3	5 ± 2	No
POPC	—	1^c^	70	100	1.8 ± 0.2	10 ± 2	No

a The discrepancies between mean molecular areas as obtained from different methodologies might be due to the hypothesis made for the adsorption isotherms, that is, the lipid mixture behaves as a single and non-ionic surfactant. In the particular case of IMM, this assumption might be inconsistent due to the different structure of the lipid species involved, such as phospholipids, CL, and cholesterol. The dissimilar surfactant properties of different lipids might result in an altered lipid composition at the interface than in the case of compression isotherms, where the lipid extract is homogeneously spread at the air-water interface.

b At π ≈ 30 mN/m.

c From *Handbook of Lipid Bilayers* by Derek Marsh. ISBN, 0849332559. CRC, press, Inc.

The compact state of OMM membranes might rely on the well-known condensing effect of cholesterol on phospholipid monolayers and bilayers (Standish and Pethica, 1967; Hung et al., 2007). Cholesterol orients the hydrocarbon chains of phospholipids perpendicularly to the plane of the bilayer and the area per molecule decreases. The condensing effect is observed in binary (Smaby et al., 1994) and ternary mixtures of lipids with cholesterol (Veatch and Keller, 2003). Moreover, cholesterol is known to interact more strongly with SM than PC through the ability of hydrogen-bond forming between the hydroxyl group of cholesterol with the amide group or the free hydroxyl group found in the sphingosine backbone of SM. Unlike PC, which presents only hydrogen bond-accepting groups, either the amide group or the free hydroxyl group can act as hydrogen bond acceptors and donors (Garcia-Arribas et al., 2016). The stronger interaction with SM seems to ground the formation of the liquid-ordered domains observed in OMM-GUVs (Figure 5) (Veatch and Keller, 2003). As expected, OMM condensed membranes have also smaller compressibility as the compressibility moduli is comparably higher than the compressibility moduli of unsaturated phospholipid monolayers (Figure 3 **)** (Wydro, 2012). Also, the presence of cholesterol and condense phases is known to increase membrane bending moduli of fluid bilayers (Arriaga et al., 2017). The bending elasticity of homogeneous OMM-GUVs reported here, $\kappa_{OMM}=7.8\pm \;2.9\;k_{B}T$ , corresponds however to rather flexible bilayers upon bending (Figure 5). Additionally, this value agrees well with the high diffusivity of RhPE within OMM supported bilayers (Figure 4). Overall, OMM lipid membranes present a condensed but fluid character that is representative for *l*_*o*_ phases.

In contrast, IMM lipids form more expanded bilayers, which results also in more fluid and more deformable membranes than OMM bilayers. The softer behaviour of IMM lipids might depend on the presence of CL among the lipid components. CL is known for its role as a modulator of membrane properties and integrity of phospholipid membranes (Nichols-Smith et al., 2004; Domenech et al., 2006; Unsay et al., 2013; Phan and Shin, 2015). The unique diphosphatidylglycerol structure of CL provides this phospholipid with four acyl chains endorsing a higher stability and the expansion of the compression isotherms on different PC/CL and PE/CL lipid monolayers (Nichols-Smith et al., 2004; Domenech et al., 2006; Phan and Shin, 2015). Their elasticity is significantly increased in consequence, as revealed by a drop in their compressibility modulus (Domenech et al., 2006; Phan and Shin, 2015). An increased lateral diffusivity is therefore compatible with this structural picture (Unsay et al., 2013), which agrees with the very expanded, fluid and deformable nature of IMM lipids found here. However, a stiffening effect in terms of the bending rigidity has been reported in DMPC bilayers by the presence of TMCL (Boscia et al., 2014). The stiffening effect relies on the rigid nature of CL as compared to PC and measured by neutron spin echo (NSE) for pure TOLC and DOPC bilayers ( $\kappa_{TOCL}\approx 26\;k_{B}T$ and $\kappa_{DOPC}\approx 18\;k_{B}T$ ) (Pan et al., 2008; Pan et al., 2015). However, the bending rigidity is highly dependent on the area per molecule of lipids and an increase of 10% in the area per acyl chain of CL makes the bending rigidity to drop a factor 2 (Doktorova et al., 2017). The effect of cholesterol on the bending stiffness is lipid-specific and the soft character of IMM-GUVs might rely on multiple factors due to the complex nature of the lipid extract, which contains a high amount of PE and cholesterol.

Real mitochondrial membranes from mammalian cells contain a high protein to phospholipid ratio (Daum and Vance, 1997). In particular, the IMM is exhibits a high protein level up to 80% w/w. Also, phospholipids are not evenly distributed across the lipid leaflets of mitochondrial membranes but rather arranged asymmetrically. Our membrane models built of lipids extracts lack both lipid asymmetry and proteins. However, the bare lipid matrix of both lipid extracts retains those interfacial properties that one would expect for highly dynamic membranes. They are essentially fluid and highly deformable upon compression and bending. These features are likely to provide mitochondrial membranes with mechanically stability and softness adequate for easy deformation in fundamental processes such us the *cristae* formation (Khalifat et al., 2008; Almendro-Vedia et al., 2021) or under the action of the fusion and fission machinery upon mitochondrial dynamics (Tolosa-Diaz et al., 2020).

## Conclusion

We show the possibility to isolate OMM and IMM from porcine heart and to extract the lipid components to generate membrane models built as lipid monolayers, supported bilayers and GUVs. A complete physicochemical characterization allowed us to obtain precise values of the mean molecular area, the CMC, diffusion coefficients and the elastic parameters upon compression and bending. Although both lipid extracts are highly deformable and fluid, OMM lipids form stiffer and more condensed membranes than IMM lipids. The membrane models composed of native lipid extracts from mitochondria open exciting opportunities to design biomimetic systems for further functional reconstitutions of mitochondrial proteins and processes.

## Data Availability

The raw data supporting the conclusion of this article will be made available by the authors, without undue reservation.

## References

[B1] Almendro-Vedia V. G. Natale P. Mell M. Bonneau S. Monroy F. Joubert F. (2017). Nonequilibrium fluctuations of lipid membranes by the rotating motor protein F1F0-ATP synthase. Proc. Natl. Acad. Sci. U. S. A. 114, 11291–11296. 10.1073/pnas.1701207114 29073046 PMC5664490

[B2] Almendro-Vedia V. Natale P. Valdivieso Gonzalez D. Lillo M. P. Aragones J. L. Lopez-Montero I. (2021). How rotating ATP synthases can modulate membrane structure. Arch. Biochem. Biophys. 708, 108939. 10.1016/j.abb.2021.108939 34052190

[B3] Arriaga L. R. Rodriguez-Garcia R. Moleiro L. H. Prevost S. Lopez-Montero I. Hellweg T. (2017). Dissipative dynamics of fluid lipid membranes enriched in cholesterol. Adv. Colloid Interface Sci. 247, 514–520. 10.1016/j.cis.2017.07.007 28755780

[B4] Axelrod D. Koppel D. E. Schlessinger J. Elson E. Webb W. W. (1976). Mobility measurement by analysis of fluorescence photobleaching recovery kinetics. Biophys. J. 16, 1055–1069. 10.1016/S0006-3495(76)85755-4 786399 PMC1334945

[B5] Beltrán-Heredia E. Tsai F. C. Salinas-Almaguer S. Cao F. J. Bassereau P. Monroy F. (2019). Membrane curvature induces cardiolipin sorting. Commun. Biol. 2, 225. 10.1038/s42003-019-0471-x 31240263 PMC6586900

[B6] Bligh E. G. Dyer W. J. (1959). A rapid method of total lipid extraction and purification. Can. J. Biochem. Physiol. 37, 911–917. 10.1139/o59-099 13671378

[B7] Bockmann R. A. Hac A. Heimburg T. Grubmuller H. (2003). Effect of sodium chloride on a lipid bilayer. Biophys. J. 85, 1647–1655. 10.1016/S0006-3495(03)74594-9 12944279 PMC1303338

[B8] Boscia A. L. Treece B. W. Mohammadyani D. Klein-Seetharaman J. Braun A. R. Wassenaar T. A. (2014). X-ray structure, thermodynamics, elastic properties and MD simulations of cardiolipin/dimyristoylphosphatidylcholine mixed membranes. Chem. Phys. Lipids 178, 1–10. 10.1016/j.chemphyslip.2013.12.010 24378240 PMC4026202

[B9] Boyd K. J. Alder N. N. May E. R. (2017). Buckling under pressure: Curvature-based lipid segregation and stability modulation in cardiolipin-containing bilayers. Langmuir 33, 6937–6946. 10.1021/acs.langmuir.7b01185 28628337 PMC5654595

[B10] Boyd K. J. Alder N. N. May E. R. (2018). Molecular dynamics analysis of cardiolipin and monolysocardiolipin on bilayer properties. Biophys. J. 114, 2116–2127. 10.1016/j.bpj.2018.04.001 29742405 PMC5961467

[B11] Bramkamp M. (2018). Bacterial dynamin-like proteins reveal mechanism for membrane fusion. Nat. Commun. 9, 3993. 10.1038/s41467-018-06559-6 30266939 PMC6162298

[B12] Brown D. A. Sabbah H. N. Shaikh S. R. (2013). Mitochondrial inner membrane lipids and proteins as targets for decreasing cardiac ischemia/reperfusion injury. Pharmacol. Ther. 140, 258–266. 10.1016/j.pharmthera.2013.07.005 23867906

[B13] Brustovetsky N. Brustovetsky T. Jemmerson R. Dubinsky J. M. (2002). Calcium-induced cytochrome c release from CNS mitochondria is associated with the permeability transition and rupture of the outer membrane. J. Neurochem. 80, 207–218. 10.1046/j.0022-3042.2001.00671.x 11902111

[B14] Catapano E. R. Arriaga L. R. Espinosa G. Monroy F. Langevin D. Lopez-Montero I. (2011). Solid character of membrane ceramides: A surface rheology study of their mixtures with sphingomyelin. Biophys. J. 101, 2721–2730. 10.1016/j.bpj.2011.10.049 22261061 PMC3297809

[B15] Catapano E. R. Lillo M. P. Garcia Rodriguez C. Natale P. Langevin D. Monroy F. (2015). Thermomechanical transitions of egg-ceramide monolayers. Langmuir 31, 3912–3918. 10.1021/acs.langmuir.5b00229 25763506

[B16] Catapano E. R. Natale P. Monroy F. Lopez-Montero I. (2017). The enzymatic sphingomyelin to ceramide conversion increases the shear membrane viscosity at the air-water interface. Adv. Colloid Interface Sci. 247, 555–560. 10.1016/j.cis.2017.07.014 28743366

[B17] Clausell A. Garcia-Subirats M. Pujol M. Busquets M. A. Rabanal F. Cajal Y. (2007). Gram-negative outer and inner membrane models: Insertion of cyclic cationic lipopeptides. J. Phys. Chem. B 111, 551–563. 10.1021/jp064757+ 17228913

[B18] Comte J. Gautheron D. C. (1979). Preparation of outer membrane from pig heart mitochondria. Methods Enzymol. 55, 98–104. 10.1016/0076-6879(79)55013-7 223005

[B19] Comte J. Maisterrena B. Gautheron D. C. (1976). Lipid composition and protein profiles of outer and inner membranes from pig heart mitochondria. Comparison with microsomes. Biochim. Biophys. Acta 419, 271–284. 10.1016/0005-2736(76)90353-9 1247555

[B20] Corey R. A. Song W. Duncan A. L. Ansell T. B. Sansom M. S. P. Stansfeld P. J. (2021). Identification and assessment of cardiolipin interactions with *E. coli* inner membrane proteins. Sci. Adv. 7, eabh2217. 10.1126/sciadv.abh2217 34417182 PMC8378812

[B21] Coskun U. Simons K. (2011). Cell membranes: The lipid perspective. Structure 19, 1543–1548. 10.1016/j.str.2011.10.010 22078554

[B22] Crimi M. Esposti M. D. (2011). Apoptosis-induced changes in mitochondrial lipids. Biochim. Biophys. Acta 1813, 551–557. 10.1016/j.bbamcr.2010.09.014 20888373

[B23] Daste F. Sauvanet C. Bavdek A. Baye J. Pierre F. Le Borgne R. (2018). The heptad repeat domain 1 of Mitofusin has membrane destabilization function in mitochondrial fusion. EMBO Rep. 19, e43637. 10.15252/embr.201643637 29661855 PMC5989784

[B24] Daum G. Vance J. E. (1997). Import of lipids into mitochondria. Prog. Lipid Res. 36, 103–130. 10.1016/s0163-7827(97)00006-4 9624424

[B25] Doktorova M. Harries D. Khelashvili G. (2017). Determination of bending rigidity and tilt modulus of lipid membranes from real-space fluctuation analysis of molecular dynamics simulations. Phys. Chem. Chem. Phys. 19, 16806–16818. 10.1039/c7cp01921a 28627570 PMC5538590

[B26] Domenech O. Sanz F. Montero M. T. Hernandez-Borrell J. (2006). Thermodynamic and structural study of the main phospholipid components comprising the mitochondrial inner membrane. Biochim. Biophys. Acta 1758, 213–221. 10.1016/j.bbamem.2006.02.008 16556434

[B27] Dowhan W. (1997). Molecular basis for membrane phospholipid diversity: Why are there so many lipids? Annu. Rev. Biochem. 66, 199–232. 10.1146/annurev.biochem.66.1.199 9242906

[B28] El-Hafidi M. Correa F. Zazueta C. (2020). Mitochondrial dysfunction in metabolic and cardiovascular diseases associated with cardiolipin remodeling. Biochim. Biophys. Acta. Mol. Basis Dis. 1866, 165744. 10.1016/j.bbadis.2020.165744 32105822

[B29] Entezami A. A. Venables B. J. Daugherty K. E. (1987). Analysis of lipids by one-dimensional thin-layer chromatography. J. Chromatogr. 387, 323–331. 10.1016/s0021-9673(01)94535-2 3558628

[B30] Evans E. Rawicz W. (1990). Entropy-driven tension and bending elasticity in condensed-fluid membranes. Phys. Rev. Lett. 64, 2094–2097. 10.1103/PhysRevLett.64.2094 10041575

[B31] Garcia-Arribas A. B. Alonso A. Goni F. M. (2016). Cholesterol interactions with ceramide and sphingomyelin. Chem. Phys. Lipids 199, 26–34. 10.1016/j.chemphyslip.2016.04.002 27132117

[B32] Giacomello M. Pyakurel A. Glytsou C. Scorrano L. (2020). The cell biology of mitochondrial membrane dynamics. Nat. Rev. Mol. Cell Biol. 21, 204–224. 10.1038/s41580-020-0210-7 32071438

[B33] Gostimskaya I. S. Grivennikova V. G. Zharova T. V. Bakeeva L. E. Vinogradov A. D. (2003). *In situ* assay of the intramitochondrial enzymes: Use of alamethicin for permeabilization of mitochondria. Anal. Biochem. 313, 46–52. 10.1016/s0003-2697(02)00534-1 12576057

[B34] Gracia R. S. Bezlyepkina N. Knorr R. L. Lipowsky R. Dimova R. (2010). Effect of cholesterol on the rigidity of saturated and unsaturated membranes: Fluctuation and electrodeformation analysis of giant vesicles. Soft Matter 6, 1472–1482. 10.1039/b920629a

[B35] Halliwell C. M. Cass A. E. (2001). A factorial analysis of silanization conditions for the immobilization of oligonucleotides on glass surfaces. Anal. Chem. 73, 2476–2483. 10.1021/ac0010633 11403288

[B36] Horvath S. E. Daum G. (2013). Lipids of mitochondria. Prog. Lipid Res. 52, 590–614. 10.1016/j.plipres.2013.07.002 24007978

[B37] Hung W. C. Lee M. T. Chen F. Y. Huang H. W. (2007). The condensing effect of cholesterol in lipid bilayers. Biophys. J. 92, 3960–3967. 10.1529/biophysj.106.099234 17369407 PMC1868968

[B38] Khalifat N. Puff N. Bonneau S. Fournier J. B. Angelova M. I. (2008). Membrane deformation under local pH gradient: Mimicking mitochondrial cristae dynamics. Biophys. J. 95, 4924–4933. 10.1529/biophysj.108.136077 18689447 PMC2576396

[B39] King E. J. (1932). The colorimetric determination of phosphorus. Biochem. J. 26, 292–297. 10.1042/bj0260292 16744823 PMC1260904

[B40] Kofeler H. C. Fauland A. Rechberger G. N. Trotzmuller M. (2012). Mass spectrometry based lipidomics: An overview of technological platforms. Metabolites 2, 19–38. 10.3390/metabo2010019 24957366 PMC3901195

[B41] Longo M. L. Ly H. V. (2007). Micropipet aspiration for measuring elastic properties of lipid bilayers. Methods Mol. Biol. 400, 421–437. 10.1007/978-1-59745-519-0_28 17951750

[B42] Lopes J. L. Nobre T. M. Cilli E. M. Beltramini L. M. Araujo A. P. Wallace B. A. (2014). Deconstructing the DGAT1 enzyme: Binding sites and substrate interactions. Biochim. Biophys. Acta 1838, 3145–3152. 10.1016/j.bbamem.2014.08.017 25152299

[B43] Lopez-Montero I. Arriaga L. R. Monroy F. Rivas G. Tarazona P. Velez M. (2008). High fluidity and soft elasticity of the inner membrane of *Escherichia coli* revealed by the surface rheology of model Langmuir monolayers. Langmuir 24, 4065–4076. 10.1021/la703350s 18338910

[B44] Lopez-Montero I. Arriaga L. R. Rivas G. Velez M. Monroy F. (2010). Lipid domains and mechanical plasticity of *Escherichia coli* lipid monolayers. Chem. Phys. Lipids 163, 56–63. 10.1016/j.chemphyslip.2009.10.002 19857475

[B45] Lopez-Montero I. Catapano E. R. Espinosa G. Arriaga L. R. Langevin D. Monroy F. (2013). Shear and compression rheology of Langmuir monolayers of natural ceramides: Solid character and plasticity. Langmuir 29, 6634–6644. 10.1021/la400448x 23621106

[B46] Marsh D. (1996). Lateral pressure in membranes. Biochimica Biophysica Acta - Rev. Biomembr. 1286, 183–223. 10.1016/s0304-4157(96)00009-3 8982283

[B47] Mathivet L. Cribier S. Devaux P. F. (1996). Shape change and physical properties of giant phospholipid vesicles prepared in the presence of an AC electric field. Biophys. J. 70, 1112–1121. 10.1016/S0006-3495(96)79693-5 8785271 PMC1225041

[B48] Merzlyakov M. Li E. Gitsov I. Hristova K. (2006). Surface-supported bilayers with transmembrane proteins: Role of the polymer cushion revisited. Langmuir 22, 10145–10151. 10.1021/la061976d 17107013

[B49] Michel J. P. Wang Y. X. Kiesel I. Gerelli Y. Rosilio V. (2017). Disruption of asymmetric lipid bilayer models mimicking the outer membrane of gram-negative bacteria by an active plasticin. Langmuir 33, 11028–11039. 10.1021/acs.langmuir.7b02864 28921990

[B50] Mileykovskaya E. Dowhan W. (2009). Cardiolipin membrane domains in prokaryotes and eukaryotes. Biochim. Biophys. Acta 1788, 2084–2091. 10.1016/j.bbamem.2009.04.003 19371718 PMC2757463

[B51] Nichols-Smith S. Teh S. Y. Kuhl T. L. (2004). Thermodynamic and mechanical properties of model mitochondrial membranes. Biochim. Biophys. Acta 1663, 82–88. 10.1016/j.bbamem.2004.02.002 15157610

[B52] Niggemann G. Kummrow M. Helfrich W. (1995). The bending rigidity of phosphatidylcholine bilayers - dependences on experimental-method, sample cell sealing and temperature. J. Phys. II Fr. 5, 413–425. 10.1051/jp2:1995141

[B53] Palade G. E. (1953). An electron microscope study of the mitochondrial structure. J. Histochem. Cytochem. 1, 188–211. 10.1177/1.4.188 13069686

[B54] Pan J. Cheng X. Sharp M. Ho C. S. Khadka N. Katsaras J. (2015). Structural and mechanical properties of cardiolipin lipid bilayers determined using neutron spin echo, small angle neutron and X-ray scattering, and molecular dynamics simulations. Soft Matter 11, 130–138. 10.1039/c4sm02227k 25369786

[B55] Pan J. Tristram-Nagle S. Kucerka N. Nagle J. F. (2008). Temperature dependence of structure, bending rigidity, and bilayer interactions of dioleoylphosphatidylcholine bilayers. Biophys. J. 94, 117–124. 10.1529/biophysj.107.115691 17827241 PMC2134881

[B56] Perez-Gil J. (2022). A recipe for a good clinical pulmonary surfactant. Biomed. J. 2022. 10.1016/j.bj.2022.03.001 PMC9486245 35272060

[B57] Pezeshkian W. König M. Wassenaar T. A. Marrink S. J. (2020). Backmapping triangulated surfaces to coarse-grained membrane models. Nat. Commun. 11, 2296. 10.1038/s41467-020-16094-y 32385270 PMC7210967

[B58] Phan M. D. Shin K. (2015). Effects of cardiolipin on membrane morphology: A Langmuir monolayer study. Biophys. J. 108, 1977–1986. 10.1016/j.bpj.2015.03.026 25902437 PMC4407250

[B59] Planas-Iglesias J. Dwarakanath H. Mohammadyani D. Yanamala N. Kagan V. E. Klein-Seetharaman J. (2015). Cardiolipin interactions with proteins. Biophys. J. 109, 1282–1294. 10.1016/j.bpj.2015.07.034 26300339 PMC4576322

[B60] Rappolt M. Hickel A. Bringezu F. Lohner K. (2003). Mechanism of the lamellar/inverse hexagonal phase transition examined by high resolution x-ray diffraction. Biophys. J. 84, 3111–3122. 10.1016/S0006-3495(03)70036-8 12719241 PMC1302872

[B61] Róg T. Martinez-Seara H. Munck N. Orešič M. Karttunen M. Vattulainen I. (2009). Role of cardiolipins in the inner mitochondrial membrane: Insight gained through atom-scale simulations. J. Phys. Chem. B 113, 3413–3422. 10.1021/jp8077369 19228006

[B62] Rosilio V. (2018). Chapter four - how can artificial lipid models mimic the complexity of molecule–membrane interactions? Adv. Biomembr. Lipid Self-Assembly 27, 107–146. 10.1016/bs.abl.2017.12.004

[B63] Schlame M. Ren M. (2009). The role of cardiolipin in the structural organization of mitochondrial membranes. Biochim. Biophys. Acta 1788, 2080–2083. 10.1016/j.bbamem.2009.04.019 19413994 PMC2757492

[B64] Schneider C. A. Rasband W. S. Eliceiri K. W. (2012). NIH image to ImageJ: 25 years of image analysis. Nat. Methods 9, 671–675. 10.1038/nmeth.2089 22930834 PMC5554542

[B65] Sennato S. Bordi F. Cametti C. Coluzza C. Desideri A. Rufini S. (2005). Evidence of domain formation in cardiolipin-glycerophospholipid mixed monolayers. A thermodynamic and AFM study. J. Phys. Chem. B 109, 15950–15957. 10.1021/jp051893q 16853024

[B66] Smaby J. M. Brockman H. L. Brown R. E. (1994). Cholesterol's interfacial interactions with sphingomyelins and phosphatidylcholines: Hydrocarbon chain structure determines the magnitude of condensation. Biochemistry 33, 9135–9142. 10.1021/bi00197a016 8049216 PMC4022348

[B67] Smith A. L. (1967). [13] Preparation, properties, and conditions for assay of mitochondria: Slaughterhouse material, small-scale. Methods Enzym. 10, 81–86. 10.1016/0076-6879(67)10016-5

[B68] Sousa J. S. D'Imprima E. Vonck J. (2018). Mitochondrial respiratory chain complexes. Subcell. Biochem. 87, 167–227. 10.1007/978-981-10-7757-9_7 29464561

[B69] Srere P. A. Sumegi B. (1986). Organization of the mitochondrial matrix. Adv. Exp. Med. Biol. 194, 13–25. 10.1007/978-1-4684-5107-8_2 3529854

[B70] Standish M. M. Pethica B. A. (1967). Interactions in phospholipid-cholesterol mixed monolayers at the air-water interface. Biochim. Biophys. Acta 144, 659–665. 10.1016/0005-2760(67)90054-9 6078125

[B71] Tian A. Johnson C. Wang W. Baumgart T. (2007). Line tension at fluid membrane domain boundaries measured by micropipette aspiration. Phys. Rev. Lett. 98, 208102. 10.1103/PhysRevLett.98.208102 17677743

[B72] Tolosa-Diaz A. Almendro-Vedia V. G. Natale P. Lopez-Montero I. (2020). The GDP-bound state of mitochondrial Mfn1 induces membrane adhesion of apposing lipid vesicles through a cooperative binding mechanism. Biomolecules 10, E1085. 10.3390/biom10071085 32708307 PMC7407159

[B73] Unsay J. D. Cosentino K. Subburaj Y. Garcia-Saez A. J. (2013). Cardiolipin effects on membrane structure and dynamics. Langmuir 29, 15878–15887. 10.1021/la402669z 23962277

[B74] van Meer G. Voelker D. R. Feigenson G. W. (2008). Membrane lipids: Where they are and how they behave. Nat. Rev. Mol. Cell Biol. 9, 112–124. 10.1038/nrm2330 18216768 PMC2642958

[B75] Veatch S. L. Keller S. L. (2003). Separation of liquid phases in giant vesicles of ternary mixtures of phospholipids and cholesterol. Biophys. J. 85, 3074–3083. 10.1016/S0006-3495(03)74726-2 14581208 PMC1303584

[B76] Vodyanoy I. Hall J. E. Balasubramanian T. M. (1983). Alamethicin-induced current-voltage curve asymmetry in lipid bilayers. Biophys. J. 42, 71–82. 10.1016/S0006-3495(83)84370-7 6838983 PMC1329204

[B77] Vyssokikh M. Y. Brdiczka D. (2003). The function of complexes between the outer mitochondrial membrane pore (VDAC) and the adenine nucleotide translocase in regulation of energy metabolism and apoptosis. Acta Biochim. Pol. 50, 389–404. 12833165

[B78] Wilson B. A. Ramanathan A. Lopez C. F. (2019). Cardiolipin-dependent properties of model mitochondrial membranes from molecular simulations. Biophys. J. 117, 429–444. 10.1016/j.bpj.2019.06.023 31349988 PMC6697365

[B79] Wydro P. (2012). Sphingomyelin/phosphatidylcholine/cholesterol monolayers--analysis of the interactions in model membranes and Brewster Angle Microscopy experiments. Colloids Surf. B Biointerfaces 93, 174–179. 10.1016/j.colsurfb.2011.12.035 22277747

[B80] Yang Z. Wang L. Yang C. Pu S. Guo Z. Wu Q. (2021). Mitochondrial membrane remodeling. Front. Bioeng. Biotechnol. 9, 786806. 10.3389/fbioe.2021.786806 35059386 PMC8763711

[B81] Yguerabide J. Schmidt J. A. Yguerabide E. E. (1982). Lateral mobility in membranes as detected by fluorescence recovery after photobleaching. Biophys. J. 40, 69–75. 10.1016/S0006-3495(82)84459-7 7139035 PMC1328974

